# Breath-Hold-Triggered BOLD fMRI in Drug-Resistant Nonlesional Focal Epilepsy—A Pilot Study

**DOI:** 10.1007/s00062-023-01363-2

**Published:** 2023-12-11

**Authors:** Christian M. Boßelmann, Josua Kegele, Leonie Zerweck, Uwe Klose, Silke Ethofer, Constantin Roder, Alexander M. Grimm, Till-Karsten Hauser

**Affiliations:** 1 Department of Neurology and Epileptology, Hertie Institute for Clinical Brain Research, University of Tuebingen, Tuebingen, Germany; 2 Department of Diagnostic and Interventional Neuroradiology, University Hospital Tuebingen, Hoppe-Seyler-Str. 3, 72076 Tuebingen, Germany; 3 Department of Neurosurgery, University Hospital Tuebingen, Tuebingen, Germany

**Keywords:** Presurgical evaluation, Epilepsy surgery, Neuroimaging, Cerebrovascular reactivity, Breath-hold

## Abstract

**Purpose:**

Individuals with drug-resistant epilepsy may benefit from epilepsy surgery. In nonlesional cases, where no epileptogenic lesion can be detected on structural magnetic resonance imaging, multimodal neuroimaging studies are required. Breath-hold-triggered BOLD fMRI (bh-fMRI) was developed to measure cerebrovascular reactivity in stroke or angiopathy and highlights regional network dysfunction by visualizing focal impaired flow increase after vasodilatory stimulus. This regional dysfunction may correlate with the epileptogenic zone. In this prospective single-center single-blind pilot study, we aimed to establish the feasibility and safety of bh-fMRI in individuals with drug-resistant non-lesional focal epilepsy undergoing presurgical evaluation.

**Methods:**

In this prospective study, 10 consecutive individuals undergoing presurgical evaluation for drug-resistant focal epilepsy were recruited after case review at a multidisciplinary patient management conference. Electroclinical findings and results of other neuroimaging were used to establish the epileptogenic zone hypothesis. To calculate significant differences in cerebrovascular reactivity in comparison to the normal population, bh-fMRIs of 16 healthy volunteers were analyzed. The relative flow change of each volume of interest (VOI) of the atlas was then calculated compared to the flow change of the whole brain resulting in an atlas of normal cerebral reactivity. Consequently, the mean flow change of every VOI of each patient was tested against the healthy volunteers group. Areas with significant impairment of cerebrovascular reactivity had decreased flow change and were compared to the epileptogenic zone localization hypothesis in a single-blind design.

**Results:**

Acquisition of bh-fMRI was feasible in 9/10 cases, with one patient excluded due to noncompliance with breathing maneuvers. No adverse events were observed, and breath-hold for intermittent hypercapnia was well tolerated. On blinded review, we observed full or partial concordance of the local network dysfunction seen on bh-fMRI with the electroclinical hypothesis in 6/9 cases, including cases with extratemporal lobe epilepsy and those with nonlocalizing 18F-fluorodeoxyglucose positron emission tomography (FDG-PET).

**Conclusion:**

This represents the first report of bh-fMRI in individuals with epilepsy undergoing presurgical evaluation. We found bh-fMRI to be feasible and safe, with a promising agreement to electroclinical findings. Thus, bh-fMRI may represent a potential modality in the presurgical evaluation of epilepsy. Further studies are needed to establish clinical utility.

**Supplementary Information:**

The online version of this article (10.1007/s00062-023-01363-2) contains supplementary material, which is available to authorized users.

## Introduction

Epilepsy affects 1–2% of people worldwide and a third of all individuals with focal epilepsy will fail to achieve seizure freedom despite treatment with two adequate and well-tolerated antiseizure medications (ASM) [1, 2]. These individuals with drug-resistant epilepsy (DRE) suffer from psychiatric and somatic comorbidities, lower economic status, social stigma, and increased all-cause mortality including sudden unexpected death in epilepsy [3–5]. Epilepsy surgery may be a safe and effective treatment option that is associated with lower morbidity, lower mortality, and a higher rate of seizure freedom when compared to prolonged medical treatment [6, 7]. The likelihood of a favorable outcome of surgical treatment is substantially higher if an epileptogenic lesion can be identified during presurgical evaluation [8]. The method of choice is structural magnetic resonance imaging (MRI) according to an epilepsy MRI protocol (HARNESS-MRI) [9, 10]. Even with advanced imaging techniques and postprocessing, MRI fails to detect a lesion in up to a third of individuals with temporal lobe epilepsy [11]. These individuals with nonlesional focal epilepsy (NLFE) may require additional testing to more clearly delineate the epileptogenic zone, including 18F-fluorodeoxyglucose (FDG) positron emission tomography (PET) or single-photon emission computed tomography (SPECT) [12].

Another imaging modality has been developed to noninvasively study cerebrovascular hemodynamics and reserve capacity in patients with ischemic stroke or moyamoya angiopathy [13–15]. Breath-hold-triggered BOLD fMRI (bh-fMRI) measures cerebrovascular reactivity, i.e., the relative change in cerebral perfusion in response to a vasodilatory stimulus [15]. Breathing maneuvers by the patient represent such a hypercapnic stimulus causing vasodilatation and thus increased cerebral blood flow. When oxygen utilization stays constant, the ratio of oxyhemoglobin to deoxyhemoglobin increases. This in turn leads to an increase in T2* BOLD signal. This method is safe, non-invasive, and does not require exposure to radiation or radionuclides. It is also cost-effective and can be acquired with few additional sequences, without the need for any additional equipment [13].

Changes in cerebral hemodynamics and reserve capacity in individuals with epilepsy are currently not well understood. Previously, Bek et al. evaluated the breath-holding index as a measure of cerebrovascular reactivity with transcranial Doppler ultrasonography (TCD) in 20 individuals with epilepsy. They noted an increased breath-holding index in the group with epilepsy when compared to healthy controls and speculated that this may be the result of an adaptive mechanism against hypoxic challenges in seizure apnea [16]. Contrarily, Chen et al. reported 81 individuals with epilepsy in which they assessed cerebral autoregulation by TCD during breath-hold maneuvers, among others. They noted a decrease in breath-holding index, autoregulation, and cerebrovascular resistance, each correlating with several measures of autonomic function, suggesting that autonomic dysfunction in individuals with epilepsy may contribute to dysregulation of cerebral blood flow [17]. More generally, Dono et al. noted that autonomic function in temporal lobe epilepsy, the most common type of focal epilepsy, may contain lateralizing information valuable for presurgical evaluation. In their study, left temporal lobe epilepsy was associated with increased cardiac vagal tone [18].

Thus, autonomic changes in cerebral hemodynamics have been proposed to highlight areas of local network dysfunction which may potentially correlate with the localization of the epileptogenic zone. Small case series of contrast-enhanced diffusion and perfusion MRI or oxygen-enhanced MRI have indeed shown potential benefit in lateralization and localization [19, 20]. Likewise, arterial-spin labeling perfusion imaging (ASL) has been demonstrated to generate findings that are concordant with PET and electroencephalography (EEG) [21, 22]. Although ASL is able to show changes in cerebral perfusion, cerebrovascular reactivity is commonly assessed by administration of a vasodilatory drug (e.g., acetazolamide) between measurements [23]. By using bh-fMRI, cerebrovascular reactivity can reliably be estimated using just a repeated short breath-hold period during a standard BOLD fMRI measurement [14]. In this prospective single-center single-blind pilot study, we aimed to establish the feasibility and safety of bh-fMRI in individuals with drug-resistant nonlesional focal epilepsy undergoing presurgical evaluation and healthy controls.

## Methods

### Cohort

This single-center study was conducted at the Department of Neurology and Epileptology, University Hospital Tübingen, between November 2021 and May 2022. Participants were prospectively and consecutively recruited from all individuals undergoing presurgical evaluation. Inclusion criteria were: i) age 18–70 years; ii) diagnosis of drug-resistant focal epilepsy according to the criteria of the Task Force of the International League Against Epilepsy (ILAE) Commission on Therapeutic Strategies [1]; iii) nonlesional focal epilepsy, i.e., prior structural imaging according to the HARNESS-MRI protocol that failed to detect an epileptogenic lesion and iv) case review at the interdisciplinary epilepsy surgery conference. Exclusion criteria were: i) inability to give informed consent; ii) any history of psychogenic nonepileptic seizures; iii) any history of primary generalized onset seizures; iv) pregnancy; v) any absolute or relative contraindications against MRI, including but not limited to presence of implanted devices, prostheses, surgical clips, wires, sutures or mesh, dental implants or retainers, or any foreign metallic body and vi) any condition that may prevent the participant from lying down for an extended period (e.g., pain, claustrophobia or pulmonary disease). Overall, 24 individuals were screened, and 10 individuals agreed to participate in the study.

### Electroclinical Hypothesis

Electroclinical data were obtained from epilepsy surgery conference reports and medical chart review. All available data were considered within the multidimensional concept of the epileptogenic zone by Lüders et al. [24]. The irritative zone, i.e., the area of cortex that generates interictal spikes, and the seizure-onset zone, i.e., the area of the cortex that initiates clinical seizures, were respectively defined with routine scalp EEG, video EEG, magnetoencephalography (MEG) or stereo electroencephalography when available. The symptomatogenic zone, i.e., the area of cortex that produces the initial ictal signs, was defined by consideration of lateralizing and localizing seizure semiology provided by the patient history or video EEG observation. The functional deficit zone, i.e., the cortical area that is functioning abnormally in the interictal period, was defined by neurological examination, neuropsychological examination, and functional imaging (18-FDG-PET-MRI) where available. Interictal SPECT was not done. Proposed localization of the epileptogenic zone was determined via blinded case review by an epileptologist who was not provided with the results of bh-fMRI before the localization hypothesis was established. The epileptogenic zone was defined as “area of cortex that is necessary and sufficient for initiating seizures and whose removal (or disconnection) is necessary for complete abolition of seizures” [25], with an additional emphasis on ictal EEG and especially confirmation by stereo electroencephalography if available [26]. This theoretical cortical zone is a primarily surgically motivated abstract concept that cannot be directly visualized on imaging, including bh-fMRI, but which represents the integrated diagnostic outcome of the presurgical evaluation. Thus, any correlation between focal abnormalities seen on bh-fMRI and this localization hypothesis would be potentially useful in a clinical setting.

### Neuroimaging and Analysis

All imaging data were acquired on a 3 T Scanner (Magnetom Prisma fit, Siemens, Erlangen, Germany) using a standard 20 channel head coil. The imaging protocol consisted of a T2* weighted EPI sequence with the following imaging parameters: TR 3000 ms, TE 36 ms, matrix 96 * 96, slice thickness 3 mm, 40 slices, FOV 245 × 245 mm, voxel size 2.6 × 2.6 × 3 mm. The paradigm consisted of 7 cycles of 9 s breath-hold in expiration followed by 60 s of normal breathing, starting with 1min of normal breathing. During the fMRI acquisition, compliance was checked via monitoring breathing using the built-in physiological monitoring capability of the scanner. The instructions were presented visually using a wall-mounted display and a coil-mounted mirror. A test run, where the participant was placed on the scanner table but outside of the gantry, preceded the final measurement to ensure that the participant understood the visually presented task. All instructions were scanner-triggered and software-generated (Presentation 20.1, Neurobehavioral Systems, Berkeley, CA, USA).

The MRI data processing was performed on a dedicated workstation. Data were preprocessed using the software package statistical parametric mapping SPM12 (The Wellcome Dept. of Imaging Neuroscience, London, UK; www.fil.ion.ucl.ac.uk/spm). After conversion into nifti file format, slice timing correction was applied to the functional data to compensate for differences in image acquisition times. The data were then realigned to remove effects caused by patient movements. After unwarping to remove geometric distortions created in regions of tissue-air interface, the functional data were co-registered with the anatomic T1 3D dataset. The data were spatially normalized to the standard Montreal Neuroimaging Institute (MNI) template, and all data were written in MNI space for subsequent evaluation.

Further analysis was done by a custom script programmed in MATLAB R2021a (The MathWorks Inc, Natick, MA, USA; http://www.mathworks.com). To improve signal quality and reduce noise, the signal was averaged using 116 volumes of interest provided by the automatic anatomic labeling atlas AAL 1 [27]. After detrending to remove signal drift during the measurement, the signal of each breath-hold period was averaged resulting in on signal time curve per volume of interest (VOI). Breath-hold periods were excluded if the cerebellar signal curve, which is assumed to be independent of supratentorial pathology [15], showed no stimulus-related positive signal curves. Furthermore, the signal time course of the cerebellar signal can be used as an independent surrogate parameter for arterial CO_2_ fluctuations, which serves to confirm physiological hemodynamic response (i.e., sufficient vasodilation after breath hold) [13, 28], including patient compliance with the breath-hold maneuvers.

Relative signal change was then calculated for each time point. Mean relative signal was calculated for the time period of positive flow response to the breath-hold maneuver and displayed in transversal sections covering all supratentorial and infratentorial structures. Random activation of physiological resting state networks may explain regional alterations of the BOLD signal. To calculate significant differences in cerebrovascular reactivity in comparison to the normal population, breath-hold fMRIs of 16 healthy volunteers were analyzed. This reference cohort had no contraindications to fMRI and no clinically significant medical conditions. The relative flow change of each VOI of the atlas was then calculated compared to the flow change of the whole brain by calculating ratios between local and global signal changes. This resulted in an atlas of normal cerebral reactivity. Consequently, the mean relative flow change of every VOI of each patient was tested against the healthy control group using Student’s independent t‑test (*p* < 0.001). The areas with significant impairment of cerebrovascular reactivity were compared to the clinical hypothesis. The neuroradiologist was blinded to all electroclinical data and was not provided with a localization hypothesis.

### Concordance

Concordance between the electroclinical localization hypothesis and the results of breath-hold fMRI was defined as such: i) full concordance corresponded to correct lobar localization and lateralization; ii) partial concordance corresponded to correct lateralization; iii) discordance corresponded to incorrect localization and lateralization. We note that it is currently unclear if breath-hold fMRI offers sufficient spatial resolution to consistently enable sublobar localization. Instead, we chose the broader concepts of lobar localization and lateralization as our endpoints, as they are of key importance in the presurgical evaluation of epilepsy and represent information with an immediate impact on surgical decision-making and a strong correlation to postoperative outcome [29–32]. This study was designed as a proof-of-concept study to establish the feasibility and safety of this novel method and was therefore not sufficiently powered for statistical analysis. Cohort size was restricted to 10 participants by the local ethics committee.

## Results

We recruited 10 participants with drug-resistant focal epilepsy syndromes. Of these, 7/10 participants presented with temporal lobe epilepsy, which represents two thirds of focal epilepsy syndromes that are assessed in tertiary epilepsy centers [33]. Seizure severity was generally high, with a mean seizure frequency of 49.6 per month (range 0.1–300, SD 87.2) and treatment failure with an average of 5.4 previous and current ASMs (range 2–10, SD 2.8). Complete electroclinical data of phase I presurgical evaluation, including seizure semiology and ictal scalp video EEG monitoring as well as neuropsychological examination results were available for all participants. Interictal and ictal EEG recordings for each participant are shown in the Supplementary Information. Other ancillary testing included magnetoencephalography (*n* = 3), PET (*n* = 6), and stereo encephalography (*n* = 3).

**Fig. 1:**
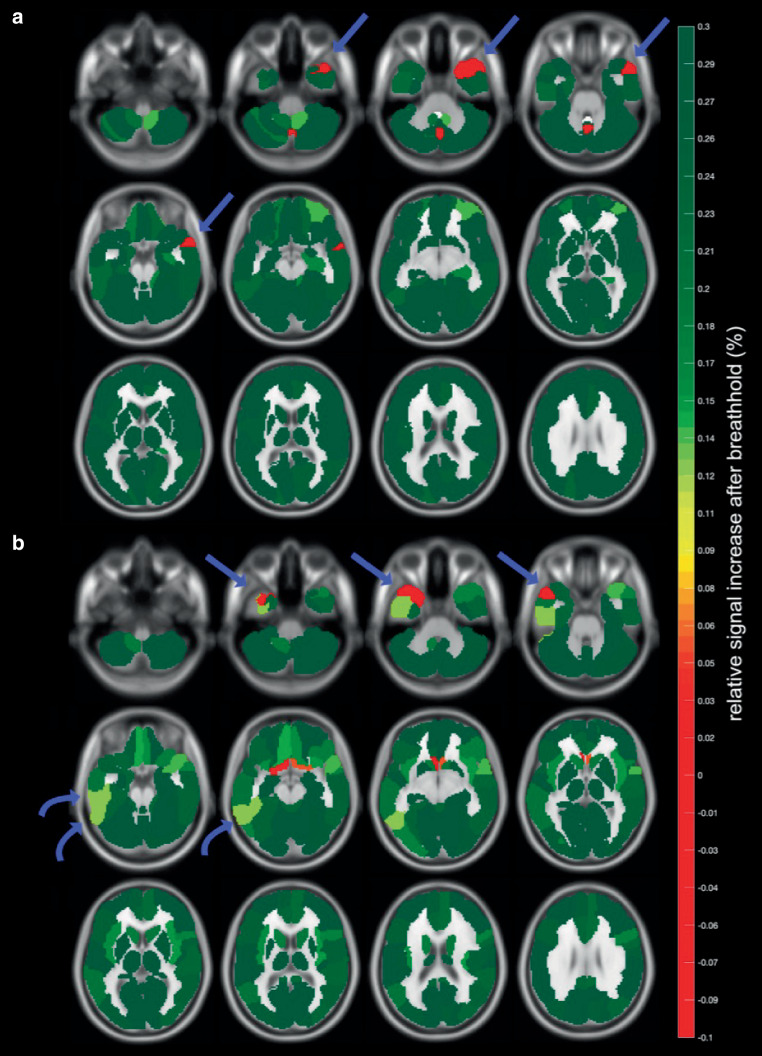
Exemplary normalized cerebrovascular reactivity (*CVR*) maps, shown as twelve representative axial slices for each study participant. **a** Participant 8. **b** Participant 6. Images are color-coded to represent the relative change in BOLD fMRI signal after breath-hold maneuvers, with impaired cerebrovascular reactivity shown in *red*. The color scale representing cerebrovascular reactivity is shown in the plot legend. Regions of interest are labelled (*arrows*)

**Fig. 2:**
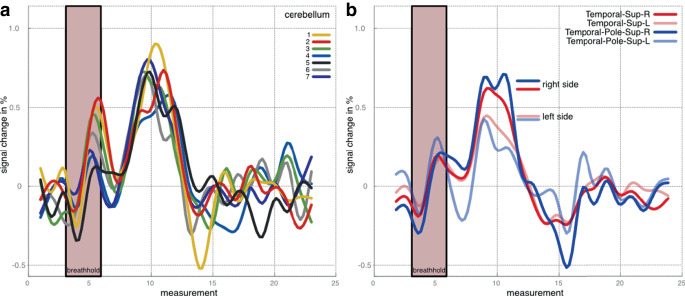
**a** Cerebellar signal curves during the 7 breath-hold periods (see color code). Note that the stimulus-invoked signal increase occurs well after the breath-hold period: The curve maximum occurs four periods (12 s) after the end of the breath-hold period (*shaded red area*). **b** Mean curves of two anatomic labeling atlas volumes-of-interest, the superior temporal (*red*) and superior temporal pole (*blue*) regions. Note the relative signal decrease in the left superior temporal regions (*light red*, *light blue*) in a patient with left temporal lobe epilepsy (participant 1)

The acquisition of bh-fMRI was feasible in 9/10 cases. One participant with left temporal lobe epilepsy was unable to comply with the breathing maneuvers and was excluded from further analysis after inspection of the signal response curve demonstrated poor data quality. We observed no adverse events, and none of the participants reported any discomfort related to intermittent hypercapnia. All participants, except for participant 6, did not have a seizure for 24 h before bh-fMRI acquisition. Exemplary results of bh-fMRI are shown as normalized cerebrovascular reactivity (CVR) maps in Fig. 1. Fig. 1a demonstrates a focal absence of relative signal increase after breath-hold maneuvers, which corresponds to an impaired CVR (red) in the left temporal region (arrow). This is consistent with the irritative zone and seizure-onset zone of participant 8. Fig. 1b demonstrates impaired CVR primarily in the right temporal region (red, straight arrow) and secondarily in the right temporo-occipital region (light green, curved arrow) which is likewise consistent with the irritative zone and seizure onset zone of participant 6. We found the cerebellar reference signal to be reproducible across the seven breath-hold periods (Fig. 2a), with an average Pearson’s correlation coefficient of 0.814. Likewise, quantitative BOLD measurements were consistent across volumes-of-interest and highlighted regions of impaired stimulus-related signal increase, e.g., in the left superior temporal regions of participant 1 (Fig. 2b). The electroclinical features, epileptogenic zone localization hypothesis, and results of bh-fMRI for all cases are summarized in Table 1.

**Table 1:** Medical history, electroclinical features and diagnostic findings of the study cohort

*Participant ID*	1	2	3	4	5	6	7	8	9	10
*Sex, age (years)*	F, 41	F, 64	F, 29	M, 62	F, 41	F, 21	M, 18	M, 24	F, 22	M, 51
*Epilepsy syndrome*	TLE	TLE	TLE	TLE	FE	FE	TLE	TLE	FE	TLE
*Age at seizure onset (years)*	27	39	27	45	6	8	13	12	15	47
*Seizure type*	FAS, FIAS	FIAS, BTCS	FAS, FIAS, BTCS	FIAS, BTCS	FIAS, BTCS	FAS	FAS, FIAS, BTCS	FIAS	FIAS, BTCS	FAS, FIAS, BTCS
*Semiology*^*1*^	Speech arrest, oral automatisms, postictal aphasia, ATLP	Nonlocalizing	Speech arrest, oral automatisms, postictal aphasia, ATLP	Oral automatisms	Right-side hyperkinetic movement, painful orofacial sensation, salivation	Nonlocalizing	Fear, tachycardia	Nonlocalizing	Right-side facial clonic and head version, postictal aphasia	Speech arrest, manual automatisms
*Seizure frequency*^*2*^	14/month	3/day	6/month	20/month	39/month	300/month	20/month	1/year	4/month	3/month
*ASM, previous*	LCM, LEV, LTG, PER	LCM, LTG, PER	LEV	LCM, LEV, LTG, ESL, PHT, TPM, VPA	CBZ, LCM, LEV, LTG, PER, PHT, PRM, VPA	LEV, OXC	BRV, LCM, LEV, OXC	OXC	BRV, LCM, LEV, LTG, ESL, OXC, PER, TPM, ZNS	LEV, PER
*ASM, current*	CNB, ESL	LTG, PRM	LTG	CNB	BRV, TPM	LEV, LTG	LCM, VPA	LTG, VPA	CNB, PER	LCM, PER
*Non-medical treatment*	None	None	None	None	KD	None	None	None	None	None
*vEEG*	Left temporal	Right temporal	Left temporal	Left hippocampal	Left posterior insula^†^	Right temporo-occipital	Right temporal^†^	Left temporal	Left insula^†^	Left temporal
*MEG*	Left temporal and frontobasal	n. d.	n. d.	n. d.	Left posterior insula, left centroparietal region	n. d.	Right frontotemporal	n. d.	n. d.	n. d.
*MRI*	Nonlesional	Nonlesional	Nonlesional	Nonlesional	Nonlesional; VBM: left insula, left lateral temporal lobe	Nonlesional	Nonlesional	Nonlesional	Nonlesional	Nonlesional
*PET*	Left temporal	n. d.	Nonlesional	n. d.	Nonlesional	n. d.	Right temporal	n. d.	Nonlesional	Nonlesional
*Neuropsychology*^*3*^	Normal	Right temporal	Impaired figural long-term memory	Left temporal	Non-lateralized frontotemporal dysfunction	Normal	Normal	Left hemisphere	Mild executive deficit	Left frontotemporal
*Localization hypothesis*	Left temporal	Right temporal	Left temporal	Left hippocampal	Left posterior insula	Right temporo-occipital	Right temporal	Left temporal	Left insula	Left temporal
*bh-fMRI*	Left temporal	Right and left temporal	Excluded	Diffuse bihemispheric	Left insula, left mesial temporal	Right temporo-occipital	Right temporal, right frontolateral	Left temporal	Left temporal, left frontal	Right temporal
*Concordance*^*4*^	++	−	0	−	++	++	++	++	+	−

*ATLP* asymmetric tonic limb posturing, *ASM* antiseizure medication, *BTCS* focal to bilateral tonic-clonic seizure, *FAS* focal-aware seizure, *FE* focal epilepsy, other than temporal lobe epilepsy, *FIAS* focal impaired awareness seizure, *MEG* magnetoencephalography, *n.* *d.* not done, *TLE* temporal lobe epilepsy, *VBM* voxel-based morphometry, *vEEG* video EEG monitoring. Antiseizure medication abbreviations: *BRV* brivaracetam, *CBZ* carbamazepine, *CNB* cenobamate, *ESL* eslicarbazepine, *KD* ketogenic diet, *LTG* lamotrigine, *LEV* levetiracetam, *LCM* lacosamide, *OXC* oxcarbazepine, *PER* perampanel, *PHT* phenytoin, *PRM* primidone, *TPM* topiramate, *VPA* valproate, *ZNS* zonisamide

^1^Lateralizing or localizing clinical signs only, not in order of evolution

^2^Average seizure frequency at time of MRI acquisition

^3^Abbreviated to lateralization or localization of dysfunction

^4^++ full concordance, + partial concordance, − discordance, 0 excluded from analysis

^†^Confirmed by stereo electroencephalography

**Fig. 3:**
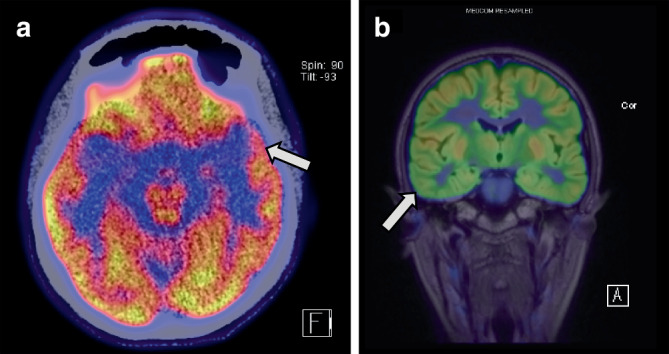
**a** FDG-PET/CT of participant 1, axial view. Statistical comparison demonstrated hypometabolism in the left temporal pole (−12%, *arrow*), left orbitofrontal cortex (−9%) and left insula (−5%). **b** FDG-PET/MRI of participant 7 demonstrating focal hypometabolism of the right lateral inferior and middle temporal gyrus (*arrow*), coronal view

In 6/9 cases, bh-fMRI showed full or partial concordance to the localization hypothesis. In two cases with left and right temporal lobe epilepsy, respectively, bh-fMRI was concordant with FDG-PET (Fig. 3). In a further two cases with left insular epilepsy (participants 5 and 9), bh-fMRI outperformed FDG-PET, which did not yield evidence of focal hypometabolism in both cases. The bh-fMRI mismatches were correctly localized to the temporal lobe, but with contralateral or bilateral lateralization (participants 2, 10). However, bh-fMRI also appropriately identified focal abnormalities in an individual with extratemporal lobe epilepsy (participant 6). In participant 4, the impairment of cerebrovascular reactivity was found to be diffusely distributed across both hemispheres.

The median length of follow-up was 329.5 days (Interquartile range, IQR, 295.5). Two participants were lost to follow-up. Participant 7 had received a hippocampus-sparing resection in the right temporal lobe without an appreciable change in seizure frequency. Of the participants 2 (1, 2) did not proceed to surgery due to later evidence suggesting bilateral seizure onset, 3 participants (3, 6, 10) achieved seizure-freedom with medical treatment and did not proceed to surgery and 2 participants (4, 8) are currently undergoing phase II evaluation. Finally, 2 participants (5, 9) were offered resective surgery but declined.

## Discussion

In this prospective single-center single-blind pilot study, bh-fMRI in patients with drug-resistant nonlesional focal epilepsy undergoing presurgical evaluation was feasible and safe. This preliminary study was not designed to establish diagnostic efficacy or clinical utility. However, we note a promising degree of agreement between the electroclinical hypothesis and bh-fMRI results in 6/9 participants. This concordance between different diagnostic modalities has previously been demonstrated to be associated with an improved surgical outcome after FDG-PET, SPECT, SPECT-MRI (SISCOM), ASL, MEG, high-density EEG, sEEG, among others [12, 34–39]. This method of multimodal integration and co-registration has become a central aspect and institutional standard for the clinical workflow of individuals with epilepsy undergoing presurgical evaluation [40]. Due to its ease of use and implementation, bh-fMRI may represent a promising adjunct modality among these multimodal neuroimaging studies.

Prior to this study, safety concerns included the use of breathing maneuvers in individuals with epilepsy. Hyperventilation is a common and safe activation procedure used during electroencephalography to increase the diagnostic yield of the method [41]. Conversely, bh-fMRI requires short periods of hypoventilation instead. In the context of sudden unexpected death in epilepsy (SUDEP), Sainju et al. measured the hypercapnic ventilatory response by modified rebreathing in 68 individuals with epilepsy [42]. This test comprised a more severe hypercapnic stimulus than the breath-hold maneuvers in this study but was still found to be safe and well tolerated. More recently, Hampson et al. investigated fMRI BOLD activation in 10 individuals with epilepsy and found differences in brainstem hypercapnia response compared to controls, which may represent a potential imaging biomarker for SUDEP. Their vasoactive stimulus was applied by a computer controlled gas delivery system for carbon dioxide (pCO_2_ < 50 mm Hg, iso-oxygenation at 110 mm Hg), not breath-hold maneuvers as in our study, and was found to be safe and well tolerated [43]. Likewise, none of our participants experienced discomfort or seizures during bh-fMRI.

Importantly, the precise mechanism by which focal abnormalities seen on bh-fMRI are generated remains unclear, and the relative contribution of regional neuronal damage, altered interictal neuronal centrality, subclinical epileptic activity with increased activity of GABAergic interneurons, and antiseizure medication is unknown. The participants in this study had drug-resistant epilepsy and therefore received many different ASMs. This is a common limitation of fMRI studies in presurgery patients [44]. Each ASM may have a different impact on functional connectivity depending on the cumulative dose, putative mechanism of action, and polytherapy [45–48]. These studies were done in cognitive networks, where the impact of ASMs is directly associated with their cognitive side effects (e.g., topiramate). It is unclear if the same concerns apply to bh-fMRI, which is instead based on cerebrovascular reactivity, but this nonetheless limits the generalizability of our findings. Furthermore, we note that we attempted to control for latent epileptic activity by analyzing participants in the interictal period, with at least 24 h since the last seizure. We acknowledge that this may be an overly simplified approach, as the effect of epileptic activity on bh-fMRI is unclear. Lastly, as the underlying mechanism of bh-fMRI is unknown, the histopathological correlate and thus the postoperative seizure outcome associated with the focal and regional abnormalities of cerebrovascular reactivity likewise remain unclear. Thus, further work is required to establish the diagnostic efficacy, concordance with other modalities, clinical utility, and impact on presurgical decision making in a larger cohort.

### Supplementary Information

Representative interictal and ictal EEG recordings of the study participants.
