# Supplementary material for: Breath-Hold-Triggered BOLD fMRI in Drug-Resistant Nonlesional Focal Epilepsy—A Pilot Study
Source: Clin Neuroradiol. 2023 Dec 11;34(2):315–24. doi: 10.1007/s00062-023-01363-2 (PMC11130005; doi:10.1007/s00062-023-01363-2)
Supplement: Supplementary file 1 — Representative interictal and ictal EEG recordings of the study participants. [file 62_2023_1363_MOESM1_ESM.pdf]

Breath-hold-triggered BOLD fMRI in drug-resistant non-lesional focal epilepsy – a pilot study.

Supplementary Information

Representative interictal and ictal EEG recordings of the study participants. Traces are shown as longitudinal bipolar and/or common average referential montages with frequency filters of LFF 1 Hz and HFF 70 Hz, no notch filter, timebase 30 mm/s, and sensitivity 7µv/mm, unless stated otherwise

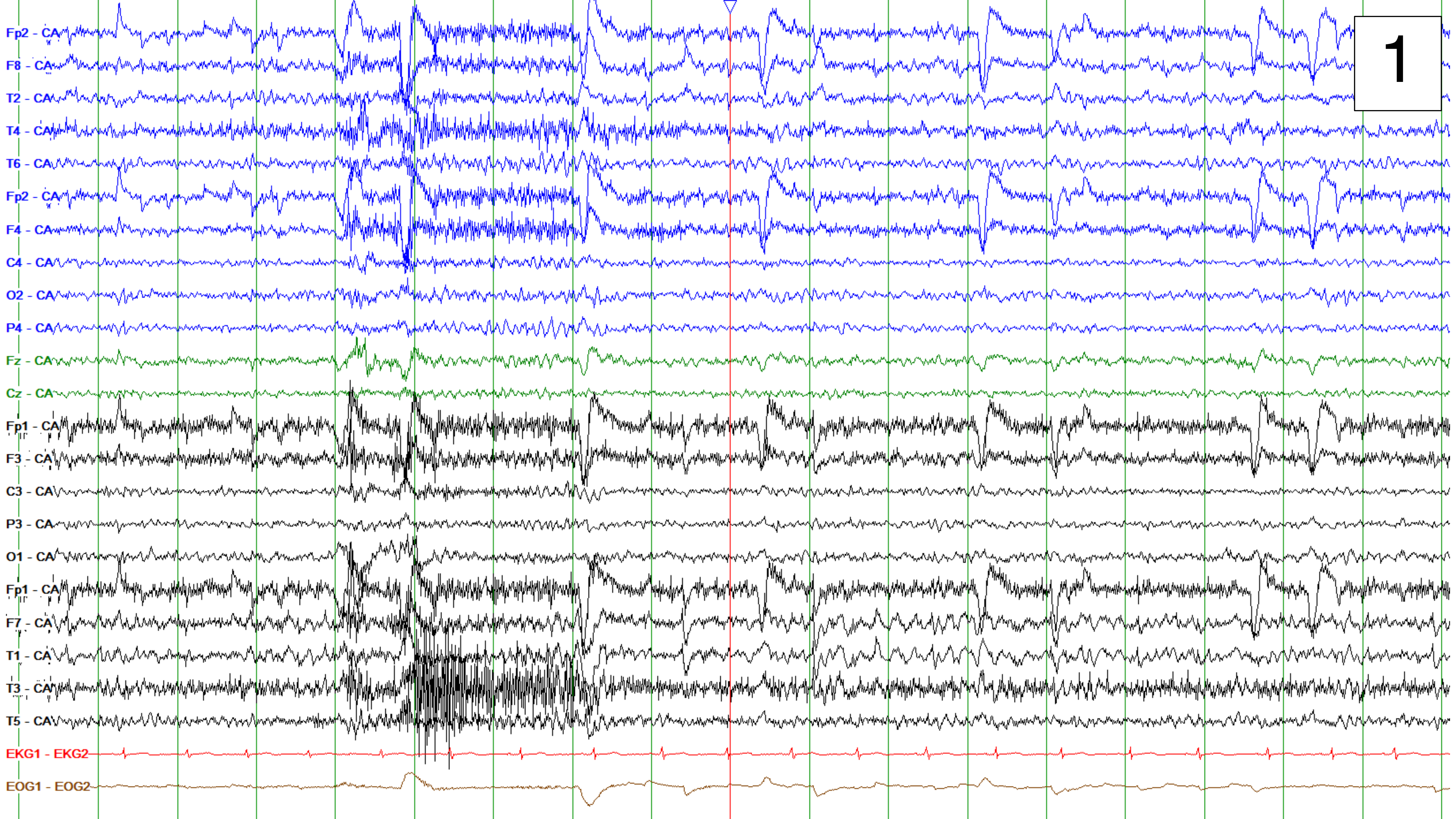
1
Fp2 - CA
F8 - CA
T2 - CA
T4 - CA
T6 - CA
Fp2 - CA
F4 - CA
C4 - CA
O2 - CA
P4 - CA
Fz - CA
Cz - CA
Fp1 - CA
F3 - CA
C3 - CA
P3 - CA
O1 - CA
Fp1 - CA
F7 - CA
T1 - CA
T3 - CA
T5 - CA
EKG1 - EKG2
EOG1 - EOG2

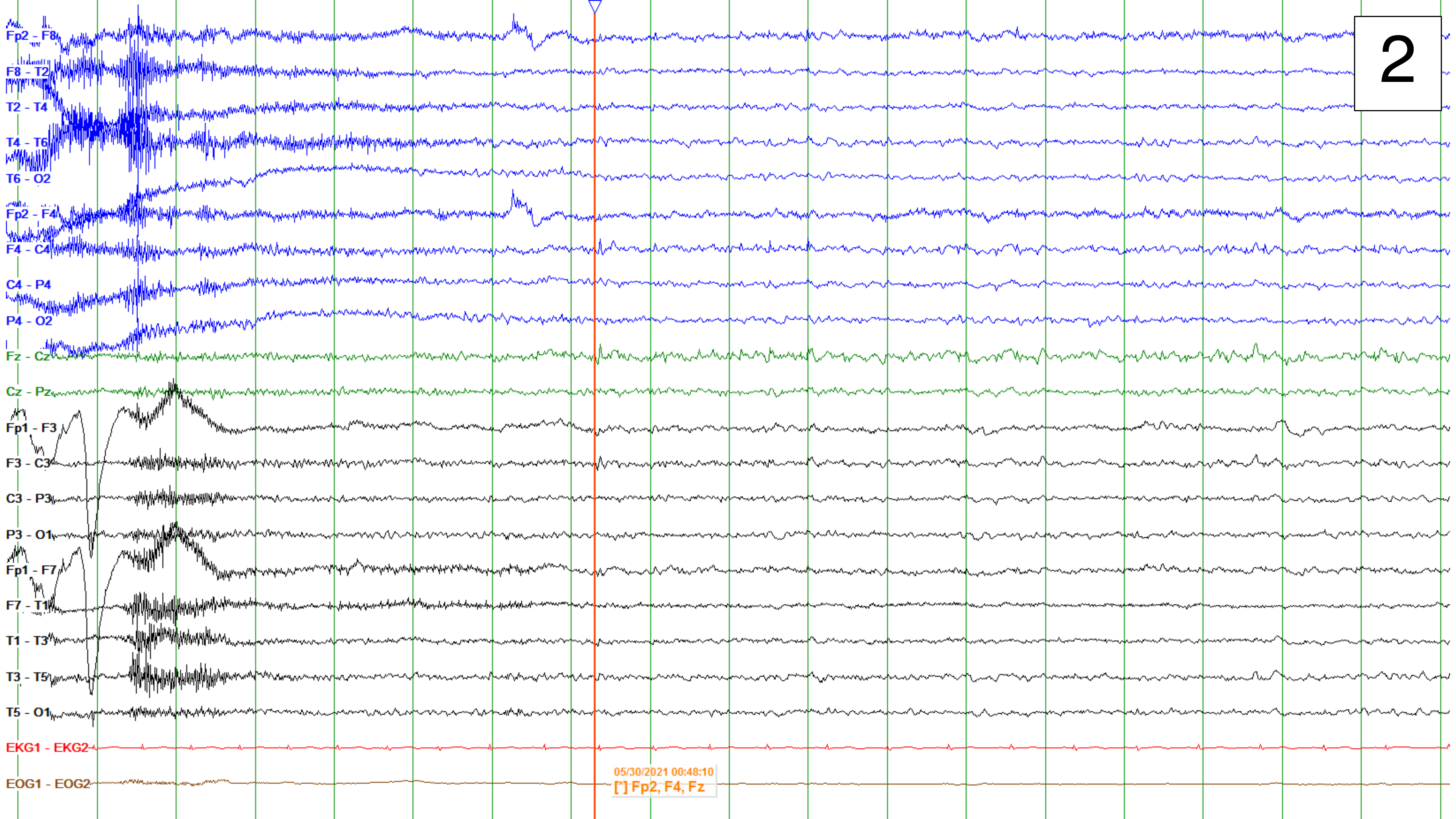
2
Fp2 - F8
F8 - T2
T2 - T4
T4 - T6
T6 - O2
Fp2 - F4
F4 - C4
C4 - P4
P4 - O2
Fz - Cz
Cz - Pz
Fp1 - F3
F3 - C3
C3 - P3
P3 - O1
Fp1 - F7
F7 - T1
T1 - T3
T3 - T5
T5 - O1
EKG1 - EKG2
EOG1 - EOG2
05/30/2021 00:48:10
[*] Fp2, F4, Fz

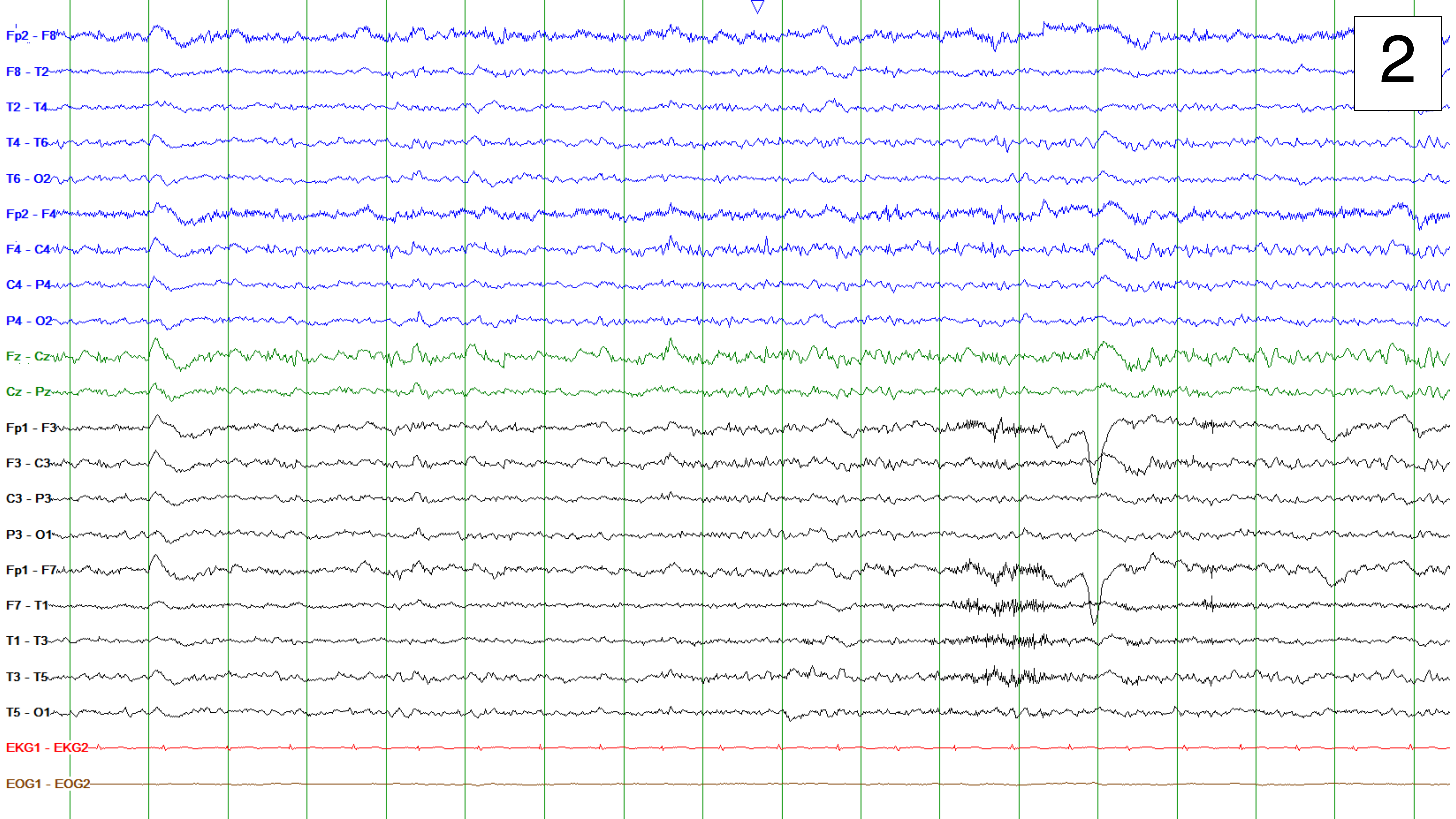
2
Fp2 - F8
F8 - T2
T2 - T4
T4 - T6
T6 - O2
Fp2 - F4
F4 - C4
C4 - P4
P4 - O2
Fz - Cz
Cz - Pz
Fp1 - F3
F3 - C3
C3 - P3
P3 - O1
Fp1 - F7
F7 - T1
T1 - T3
T3 - T5
T5 - O1
EKG1 - EKG2
EOG1 - EOG2

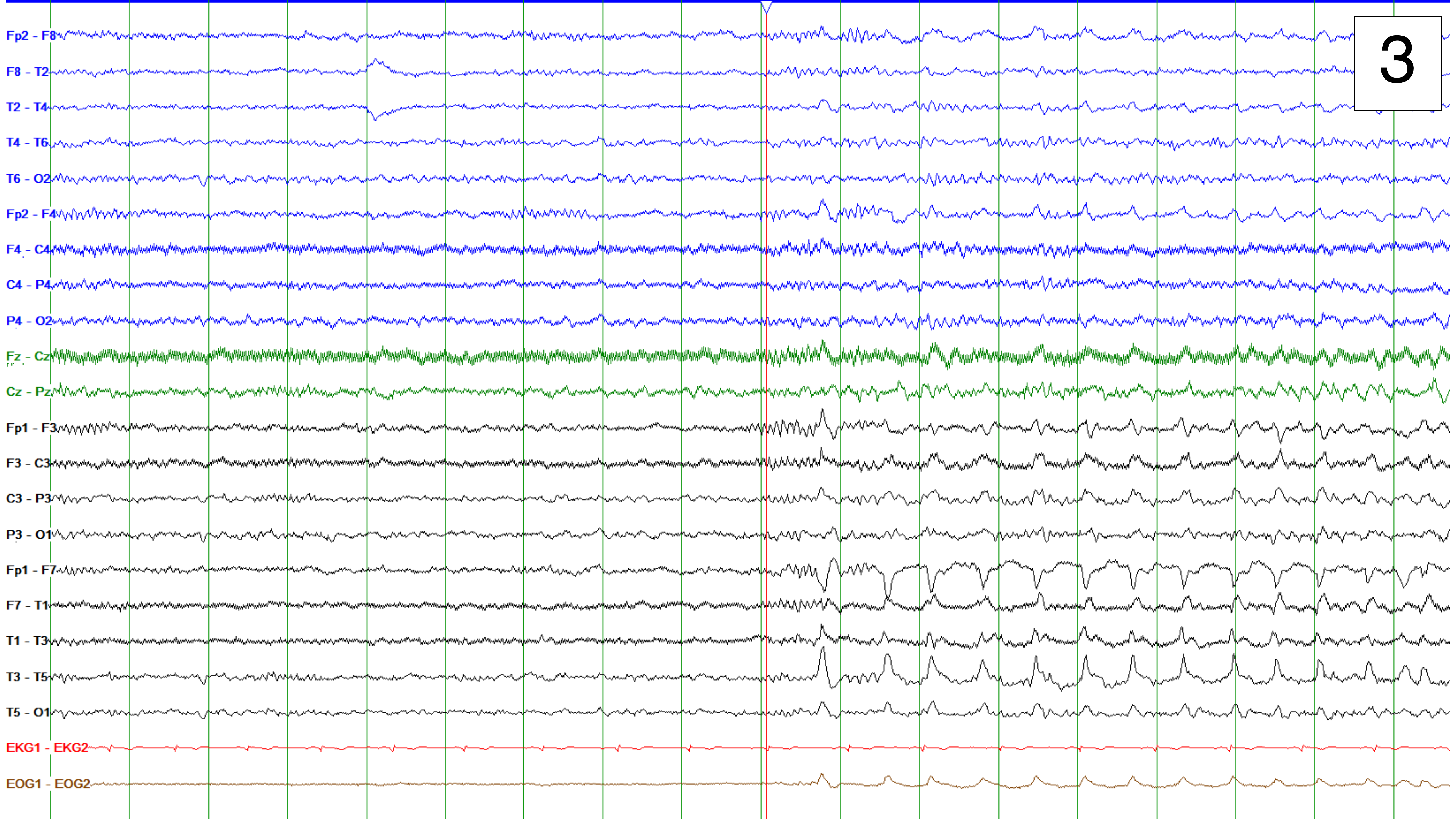
3
Fp2 - F8
F8 - T2
T2 - T4
T4 - T6
T6 - O2
Fp2 - F4
F4 - C4
C4 - P4
P4 - O2
Fz - Cz
Cz - Pz
Fp1 - F3
F3 - C3
C3 - P3
P3 - O1
Fp1 - F7
F7 - T1
T1 - T3
T3 - T5
T5 - O1
EKG1 - EKG2
EOG1 - EOG2

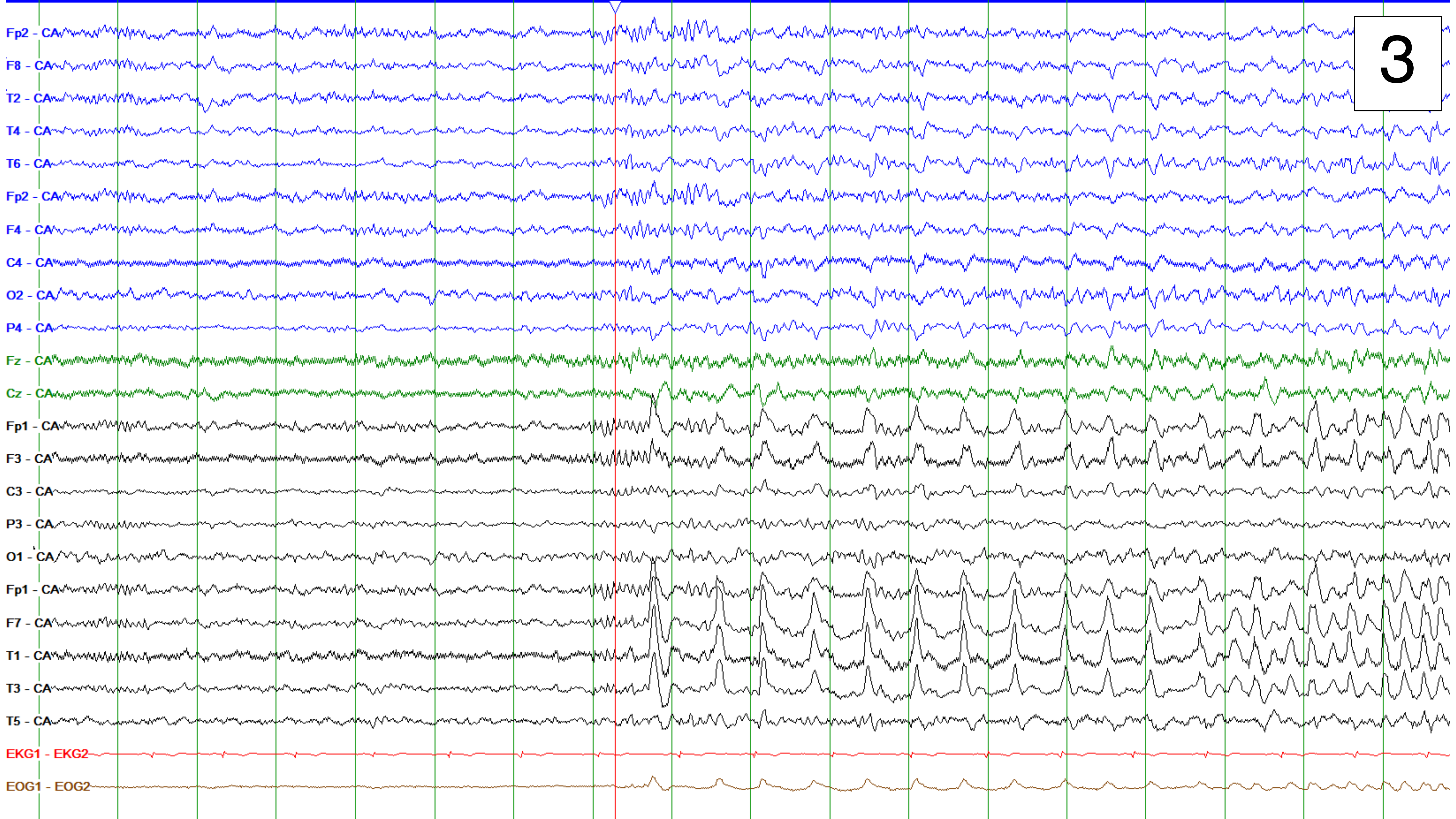
3
Fp2 - CA
F8 - CA
T2 - CA
T4 - CA
T6 - CA
Fp2 - CA
F4 - CA
C4 - CA
O2 - CA
P4 - CA
Fz - CA
Cz - CA
Fp1 - CA
F3 - CA
C3 - CA
P3 - CA
O1 - CA
Fp1 - CA
F7 - CA
T1 - CA
T3 - CA
T5 - CA
EKG1 - EKG2
EOG1 - EOG2

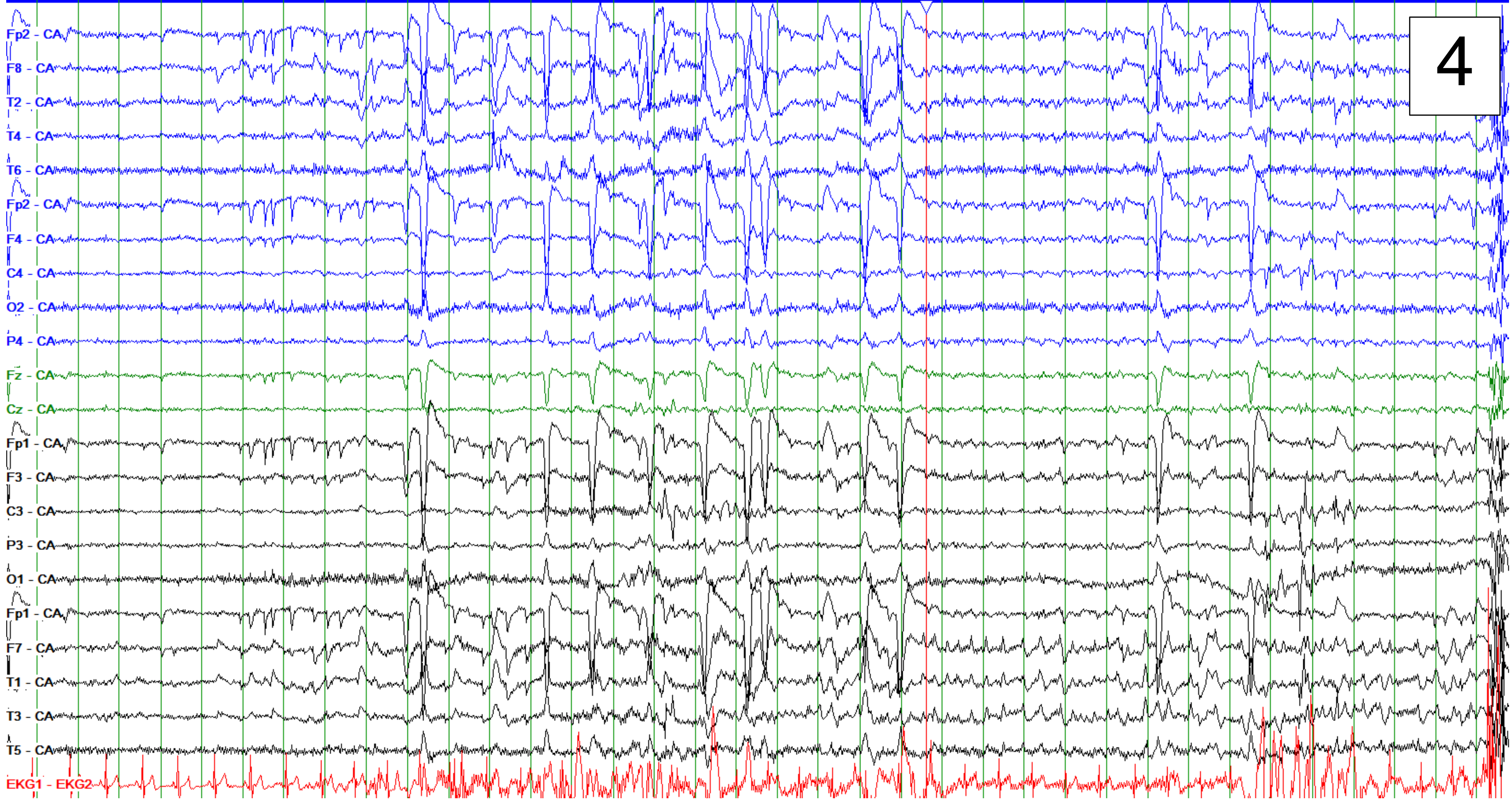

LFF 1 Hz, HFF 30 Hz, notch 50 Hz, sensitivity 10 μv/mm, timebase 15 mm/s

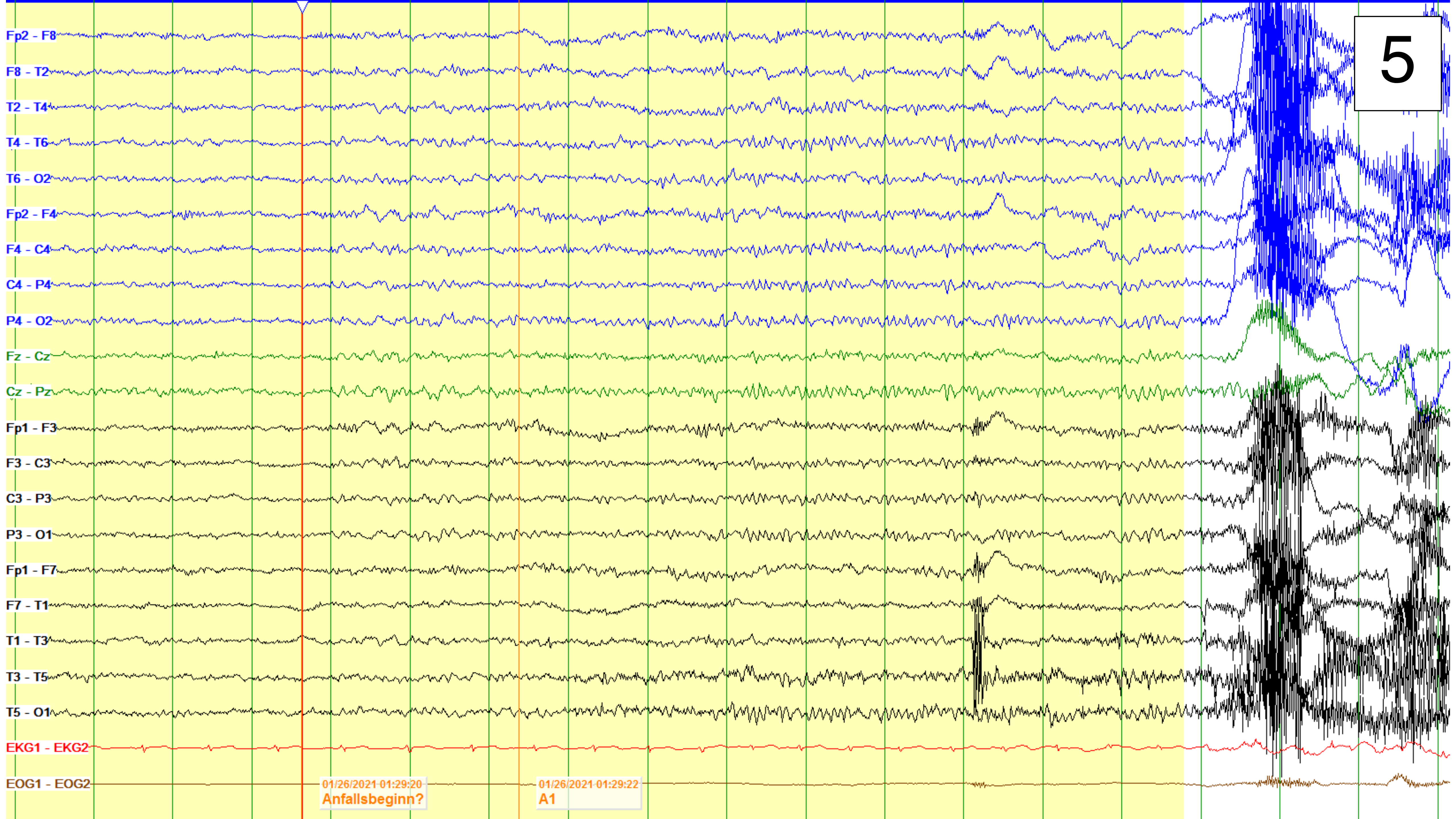
5
Fp2 - F8
F8 - T2
T2 - T4
T4 - T6
T6 - O2
Fp2 - F4
F4 - C4
C4 - P4
P4 - O2
Fz - Cz
Cz - Pz
Fp1 - F3
F3 - C3
C3 - P3
P3 - O1
Fp1 - F7
F7 - T1
T1 - T3
T3 - T5
T5 - O1
EKG1 - EKG2
EOG1 - EOG2
01/26/2021 01:29:20
Anfallsbeginn?
01/26/2021 01:29:22
A1

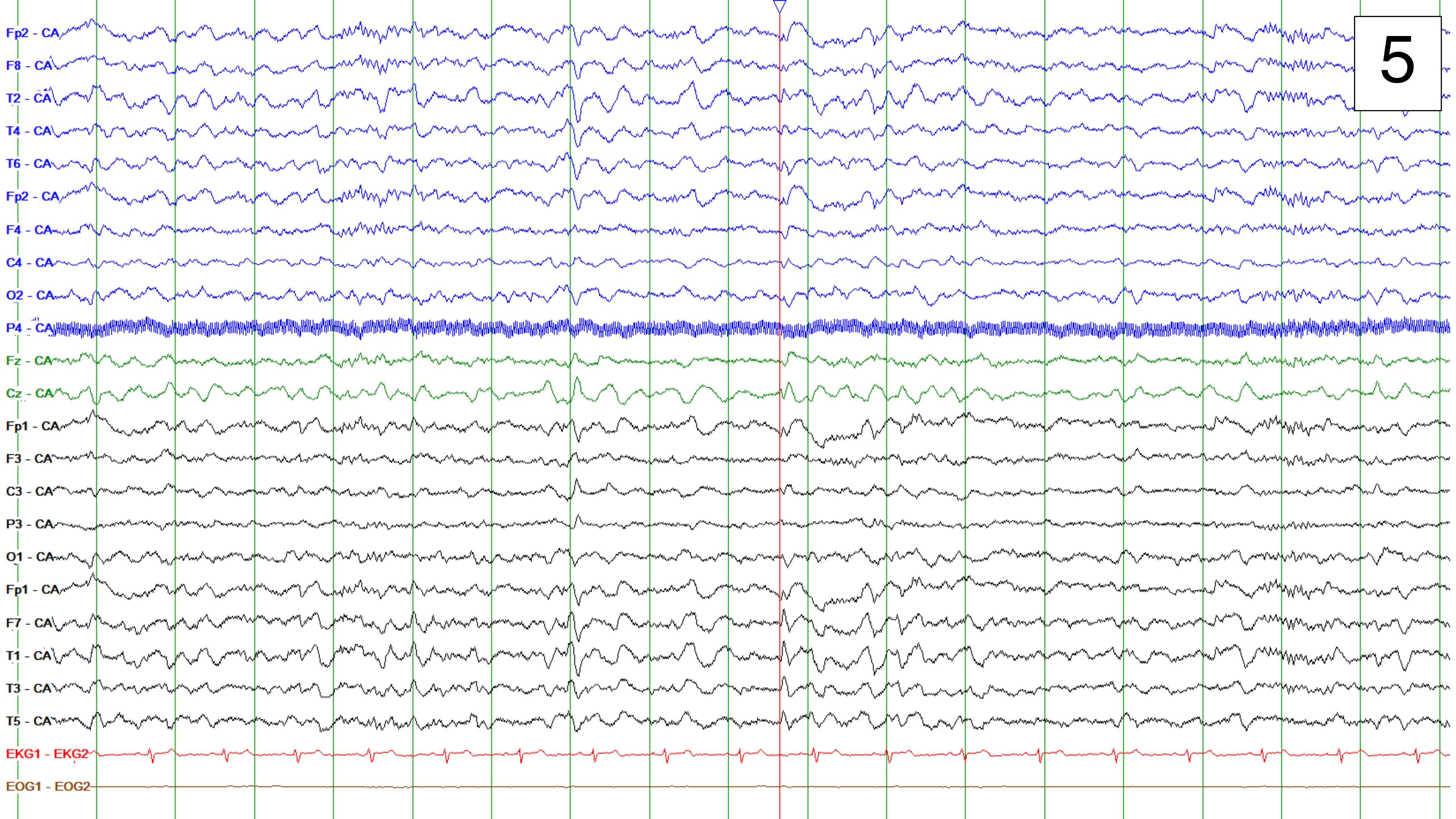
5
Fp2 - CA
F8 - CA
T2 - CA
T4 - CA
T6 - CA
Fp2 - CA
F4 - CA
C4 - CA
O2 - CA
P4 - CA
Fz - CA
Cz - CA
Fp1 - CA
F3 - CA
C3 - CA
P3 - CA
O1 - CA
Fp1 - CA
F7 - CA
T1 - CA
T3 - CA
T5 - CA
EKG1 - EKG2
EOG1 - EOG2

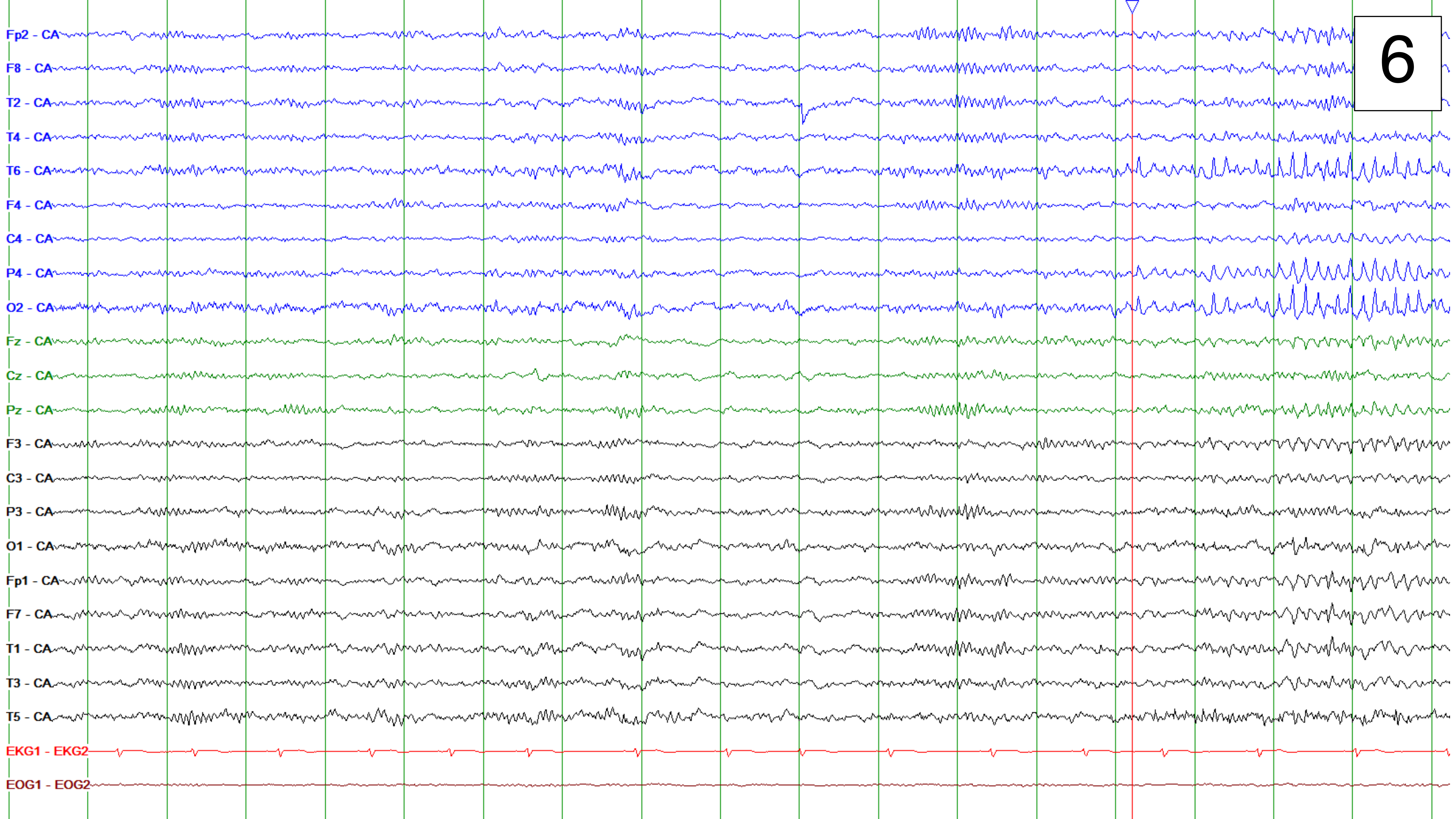
Fp2 - CA
F8 - CA
T2 - CA
T4 - CA
T6 - CA
F4 - CA
C4 - CA
P4 - CA
O2 - CA
Fz - CA
Cz - CA
Pz - CA
F3 - CA
C3 - CA
P3 - CA
O1 - CA
Fp1 - CA
F7 - CA
T1 - CA
T3 - CA
T5 - CA
EKG1 - EKG2
EOG1 - EOG2
6

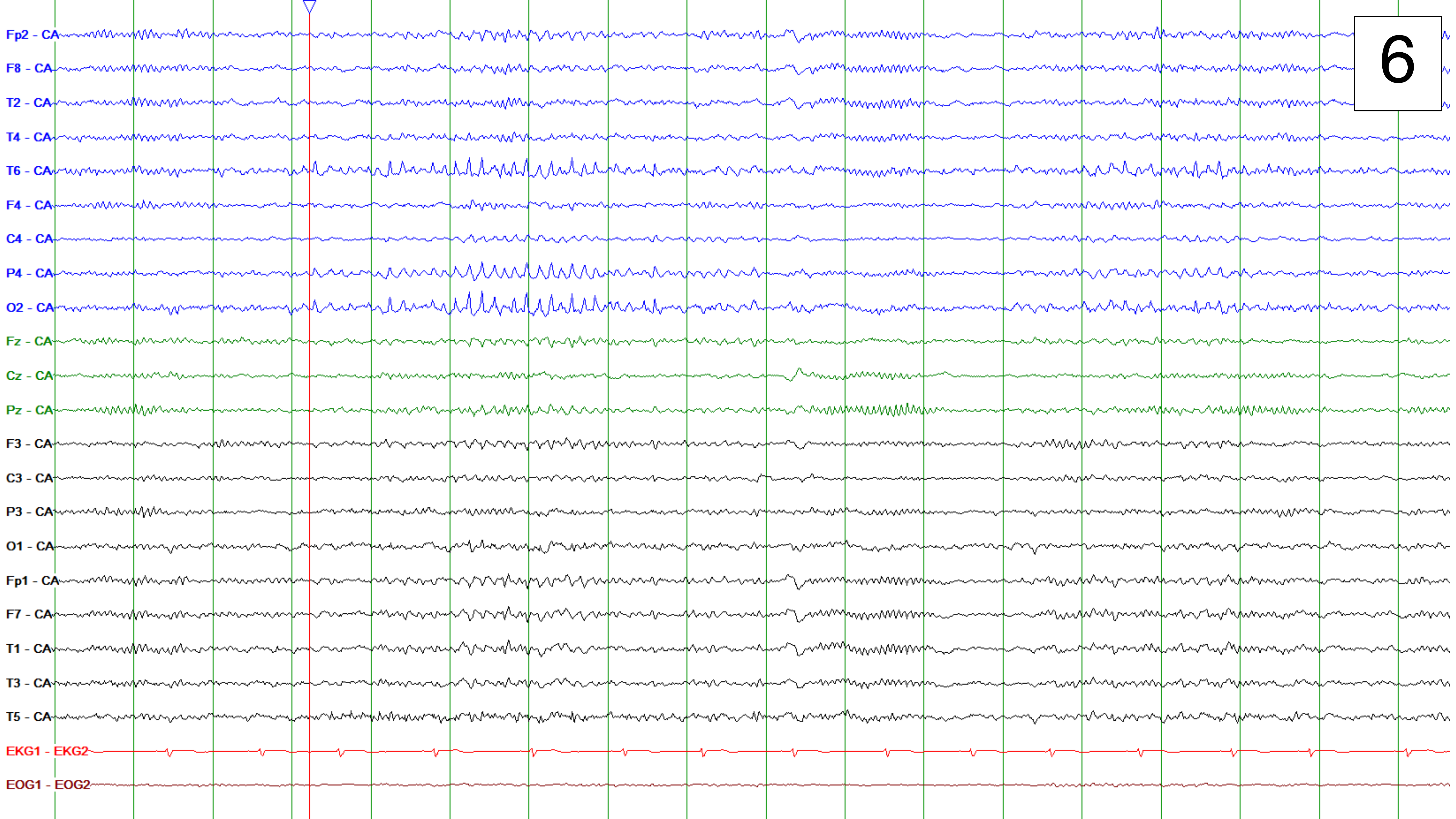
6
Fp2 - CA
F8 - CA
T2 - CA
T4 - CA
T6 - CA
F4 - CA
C4 - CA
P4 - CA
O2 - CA
Fz - CA
Cz - CA
Pz - CA
F3 - CA
C3 - CA
P3 - CA
O1 - CA
Fp1 - CA
F7 - CA
T1 - CA
T3 - CA
T5 - CA
EKG1 - EKG2
EOG1 - EOG2

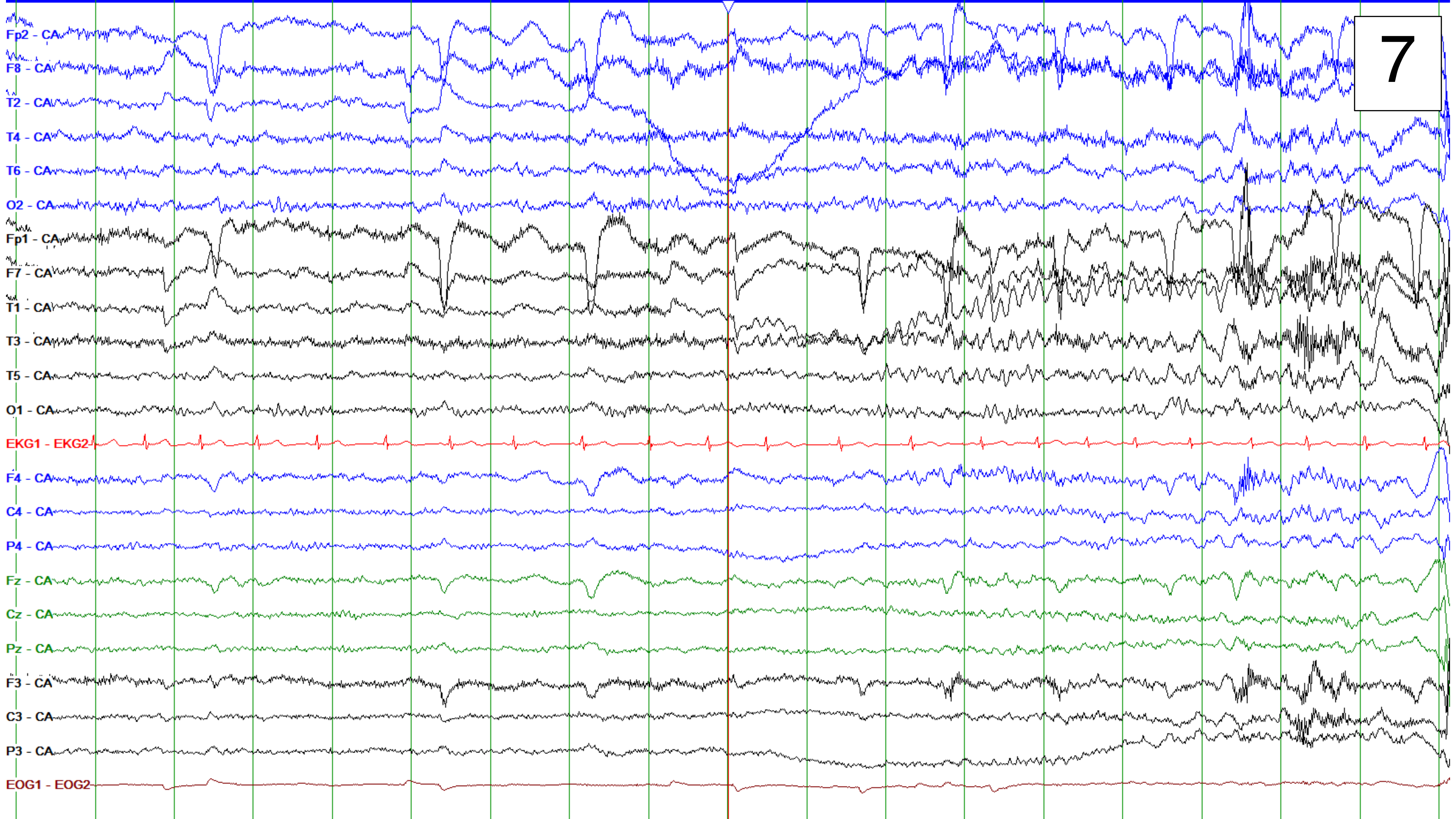

7
Fp2 - CA
F8 - CA
T2 - CA
T4 - CA
T6 - CA
O2 - CA
Fp1 - CA
F7 - CA
T1 - CA
T3 - CA
T5 - CA
O1 - CA
EKG1 - EKG2
F4 - CA
C4 - CA
P4 - CA
Fz - CA
Cz - CA
Pz - CA
F3 - CA
C3 - CA
P3 - CA
EOG1 - EOG2

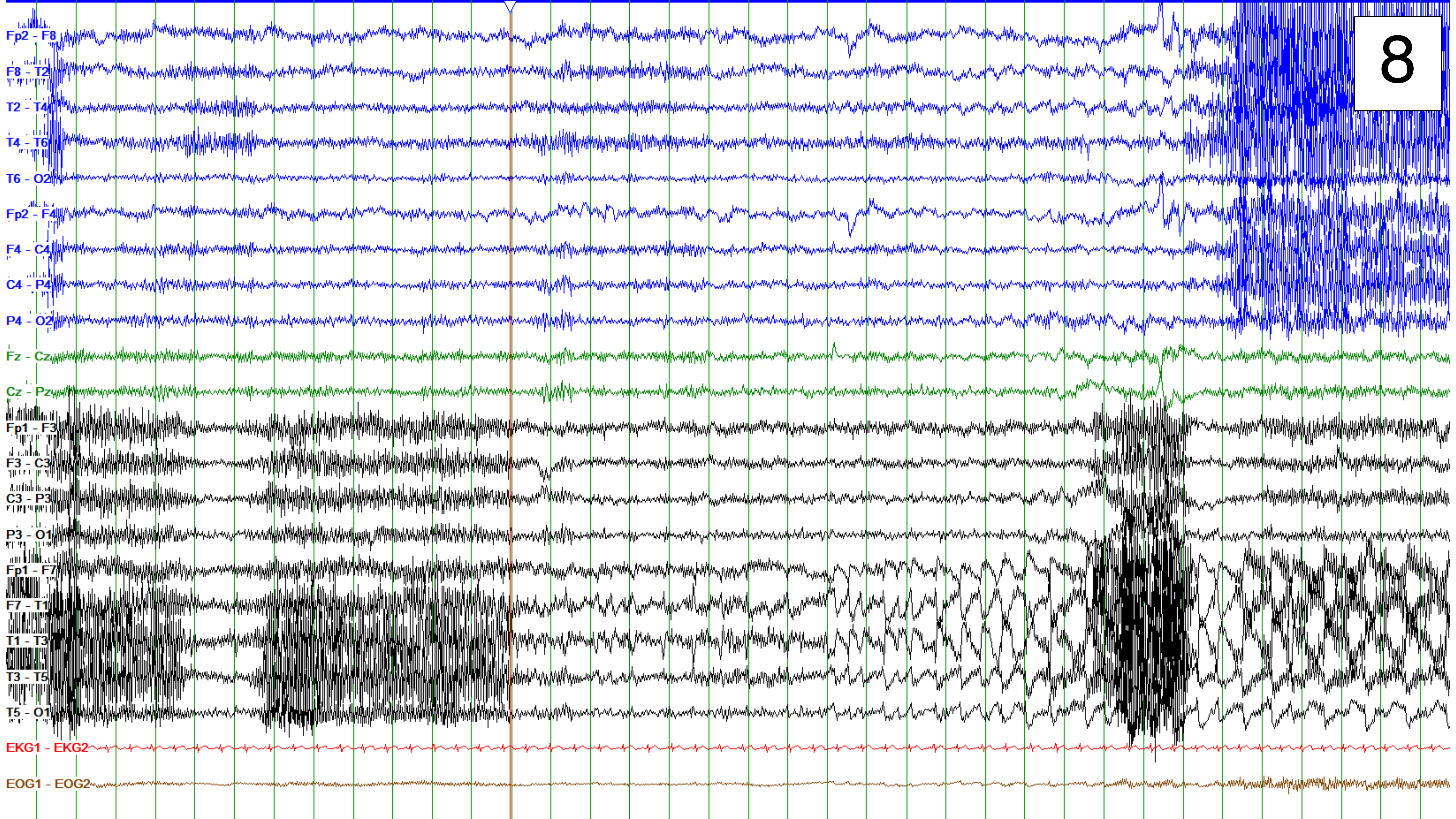
8
Fp2 - F8
F8 - T2
T2 - T4
T4 - T6
T6 - O2
Fp2 - F4
F4 - C4
C4 - P4
P4 - O2
Fz - Cz
Cz - Pz
Fp1 - F3
F3 - C3
C3 - P3
P3 - O1
Fp1 - F7
F7 - T1
T1 - T3
T3 - T5
T5 - O1
EKG1 - EKG2
EOG1 - EOG2

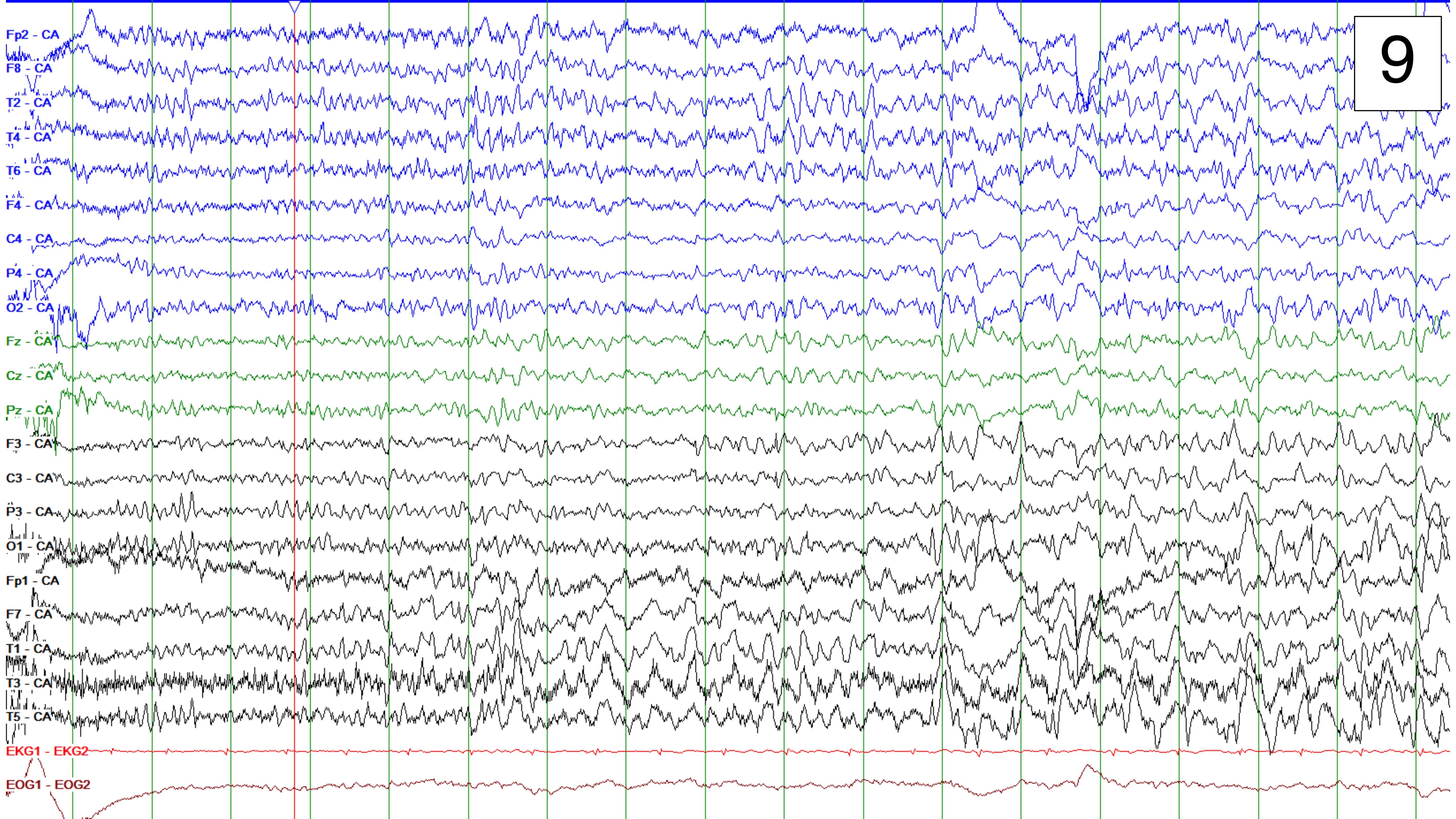
9
Fp2 - CA
F8 - CA
T2 - CA
T4 - CA
T6 - CA
F4 - CA
C4 - CA
P4 - CA
O2 - CA
Fz - CA
Cz - CA
Pz - CA
F3 - CA
C3 - CA
P3 - CA
O1 - CA
Fp1 - CA
F7 - CA
T1 - CA
T3 - CA
T5 - CA
EKG1 - EKG2
EOG1 - EOG2

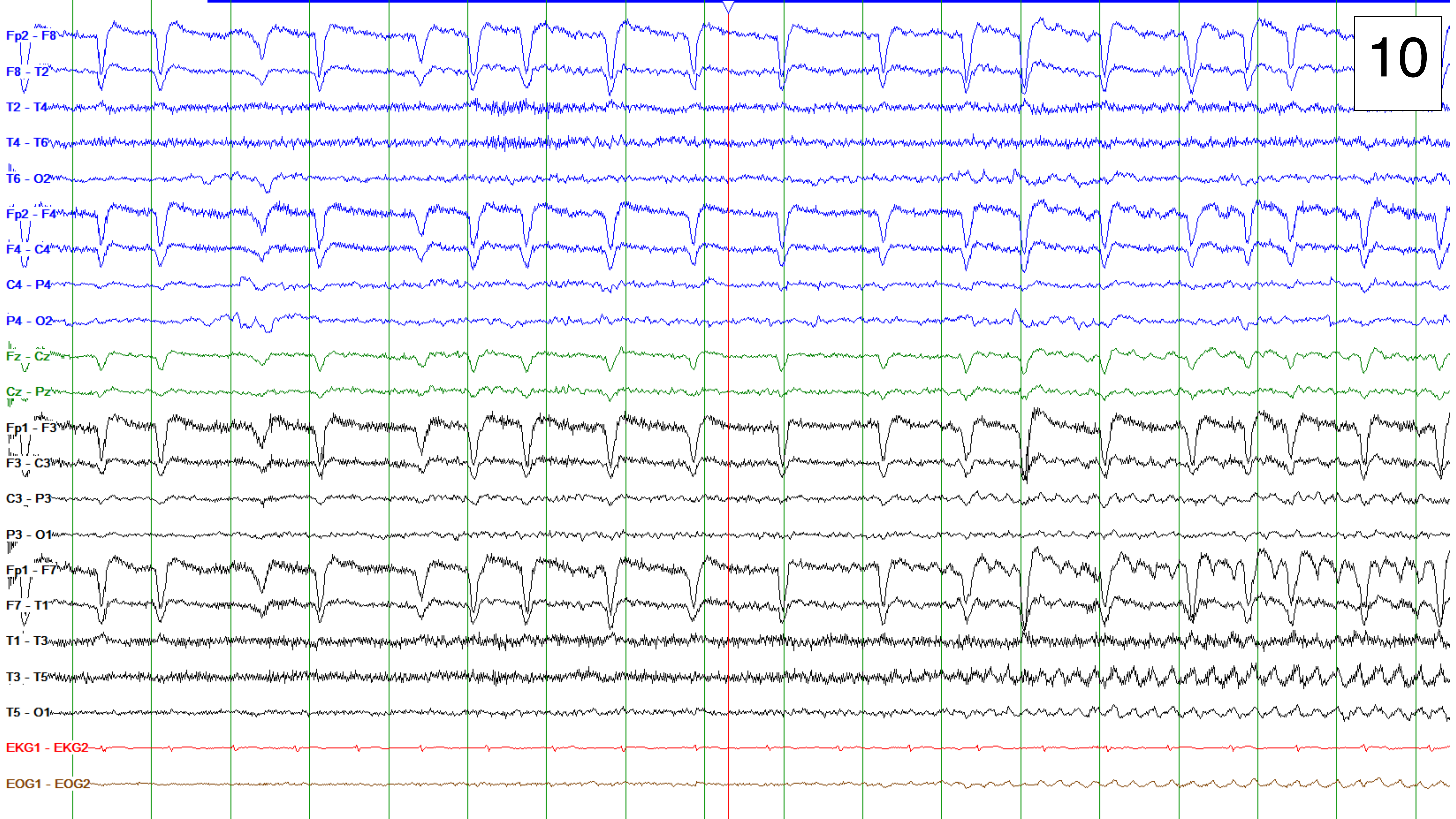
10
Fp2 - F8
F8 - T2
T2 - T4
T4 - T6
T6 - O2
Fp2 - F4
F4 - C4
C4 - P4
P4 - O2
Fz - Cz
Cz - Pz
Fp1 - F3
F3 - C3
C3 - P3
P3 - O1
Fp1 - F7
F7 - T1
T1 - T3
T3 - T5
T5 - O1
EKG1 - EKG2
EOG1 - EOG2

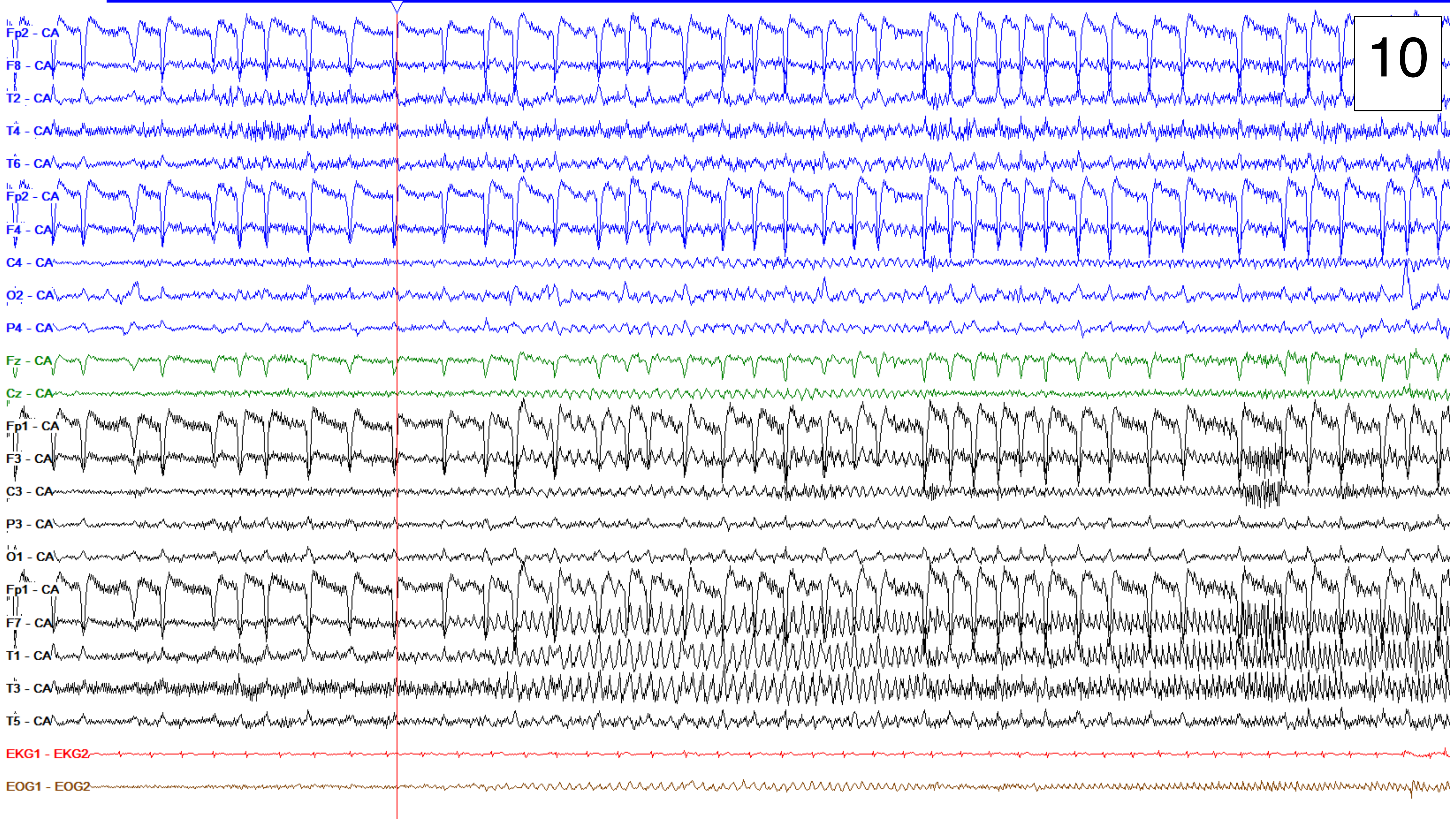
10
Fp2 - CA
F8 - CA
T2 - CA
T4 - CA
T6 - CA
Fp2 - CA
F4 - CA
C4 - CA
O2 - CA
P4 - CA
Fz - CA
Cz - CA
Fp1 - CA
F3 - CA
C3 - CA
P3 - CA
O1 - CA
Fp1 - CA
F7 - CA
T1 - CA
T3 - CA
T5 - CA
EKG1 - EKG2
EOG1 - EOG2
